# 3D osteogenic differentiation of human iPSCs reveals the role of TGFβ signal in the transition from progenitors to osteoblasts and osteoblasts to osteocytes

**DOI:** 10.1038/s41598-023-27556-w

**Published:** 2023-01-19

**Authors:** Shunsuke Kawai, Junko Sunaga, Sanae Nagata, Megumi Nishio, Masayuki Fukuda, Takeshi Kamakura, Liping Sun, Yonghui Jin, Satoko Sakamoto, Akira Watanabe, Shuichi Matsuda, Taiji Adachi, Junya Toguchida

**Affiliations:** 1 Department of Fundamental Cell Technology, Center for iPS Cell Research and Application, Kyoto University, Kyoto, Japan; 2 Department of Orthopaedic Surgery, Graduate School of Medicine, Kyoto University, Kyoto, Japan; 3 Department of Biosystems Science, Institute for Life and Medical Sciences, Kyoto University, Kyoto, Japan; 4 Department of Regeneration Sciences and Engineering, Institute for Life and Medical Sciences, Kyoto University, Kyoto, Japan; 5 Medical Innovation Center, Graduate School of Medicine, Kyoto University, Kyoto, Japan

**Keywords:** Multipotent stem cells, Differentiation, Transcriptomics, Cell invasion, Growth factor signalling

## Abstract

Although the formation of bone-like nodules is regarded as the differentiation process from stem cells to osteogenic cells, including osteoblasts and osteocytes, the precise biological events during nodule formation are unknown. Here we performed the osteogenic induction of human induced pluripotent stem cells using a three-dimensional (3D) culture system using type I collagen gel and a rapid induction method with retinoic acid. Confocal and time-lapse imaging revealed the osteogenic differentiation was initiated with vigorous focal proliferation followed by aggregation, from which cells invaded the gel. Invading cells changed their morphology and expressed osteocyte marker genes, suggesting the transition from osteoblasts to osteocytes. Single-cell RNA sequencing analysis revealed that 3D culture-induced cells with features of periosteal skeletal stem cells, some of which expressed TGFβ-regulated osteoblast-related molecules. The role of TGFβ signal was further analyzed in the transition from osteoblasts to osteocytes, which revealed that modulation of the TGFβ signal changed the morphology and motility of cells isolated from the 3D culture, suggesting that the TGFβ signal maintains the osteoblastic phenotype and the transition into osteocytes requires down-regulation of the TGFβ signal.

## Introduction

Bone is a major tissue in the skeleton. It configures the body stature and protects vital visceral organs along with contributing to the locomotive system in cooperation with muscle and nerve. Bone tissues also serve as a reservoir of minerals and support hematopoiesis. During the embryonic period, bones are built via several distinct embryonic lineages, and their developmental origin is reflected in extracellular matrix composition, structure and anatomical location^1^. Bones in the craniofacial region are mainly derived from the neural crest, axial components from paraxial mesoderm, and appendicular skeletons from lateral plate mesoderm^2^.

In vitro experiments have shown that osteochondroprogenitor cells in each of the above developmental lineages differentiate into osteoblasts, which secrete type I collagens and build bone matrix^3–5^. Postnatally, osteoblasts are supplied from skeletal stem cells (SSCs) in the periosteum or bone marrow^6^, although SSCs in the bone marrow are not clearly distinguished from bone marrow mesenchymal stem cells (MSCs)^1^. A subset of osteoblasts enter the apoptotic process, and the rest are thought to either stay on the surface of bone as inactive bone lining cells or become osteocytes that are terminally differentiated cells in the osteogenic lineage upon embedding within the bone matrix^7^. These two major osteogenic cell types have quite different characters. Osteoblasts have a large cytoplasm, are polygonal in shape and have a short lifespan, whereas osteocytes have a small cytoplasm, are dendritic in shape with many processes and have a very long lifespan^1,7^. Their biological functions are also different. In unstimulated conditions, osteoblasts localize on the surface of bone matrix as bone lining cells, which are activated by the remodeling signal, secrete bone matrix component, such as type I collagen, and form the basis of matrix mineralization^1,7^. As for osteocytes, their roles in bone metabolism have gathered much attention in recent years^8^. They act as mechanosensory cells and regulate bone formation and resorption by sensing the signal and transmitting it via soluble factors to osteoblasts and osteoclasts^9^. The transition from osteoblasts to osteocytes is not a simple process, and each cell type consists of multiple subpopulations with different phenotypes^10^. Matrix formation by osteoblasts, which modifies cell motility, has been proposed to be a key factor in this transition^10,11^. Therefore, a precise analysis of this process requires an in vitro culture system, such as a three-dimensional (3D) culture system using type I collagen gels, that mimics the physiological bone environment^12^.

The osteoblastic differentiation of rat bone marrow cells is enhanced by culture in type I collagen matrix, suggesting a role of collagen-integrin interactions^13^. Primary rat osteoblasts or MC3T3-E1, an established murine pre-osteoblast cell line^14^, cultured in 3D collagen gels demonstrated faster mineralization and a larger expression of osteoblast-related genes compared with those in two-dimensional (2D) culture using collagen-coated plates, indicating the importance of the 3D configuration of gels^15^. The migration and transition to osteocyte-like cells of MC3T3-E1 and mouse calvaria osteoblasts were observed by culturing on type I collagen gel layers with osteogenic differentiation medium^16^. These data indicate the importance of cell–matrix interactions in the transition.

To understand the differentiation process in detail, we developed an i*n vitro* culture system to observe the entire process of differentiation from pluripotent stem cells (PSCs) to osteocytes. Recently we have established an osteogenic differentiation method using retinoic acid, which induces bone like nodules containing osteoblastic and osteocytic cells from human induced pluripotent stem cell (hiPSC) within ten days^17^. Taking advantage of the rapid induction, we succeeded to visualize the process of bone-like nodule formation by time-lapse imaging and demonstrated the transition from osteoblastic cells to osteocytic cells^17^. Here we combined this method with a 3D collagen gel culture system to visualize the differentiation process from hiPSCs to osteoblasts and finally osteocytes. In addition, using single-cell RNA (scRNA) sequencing analysis, we investigated the differentiation process at the molecular level.

## Results

### Induction of osteoblastic and osteocytic cells on type I collagen gel

**Figure 1:**
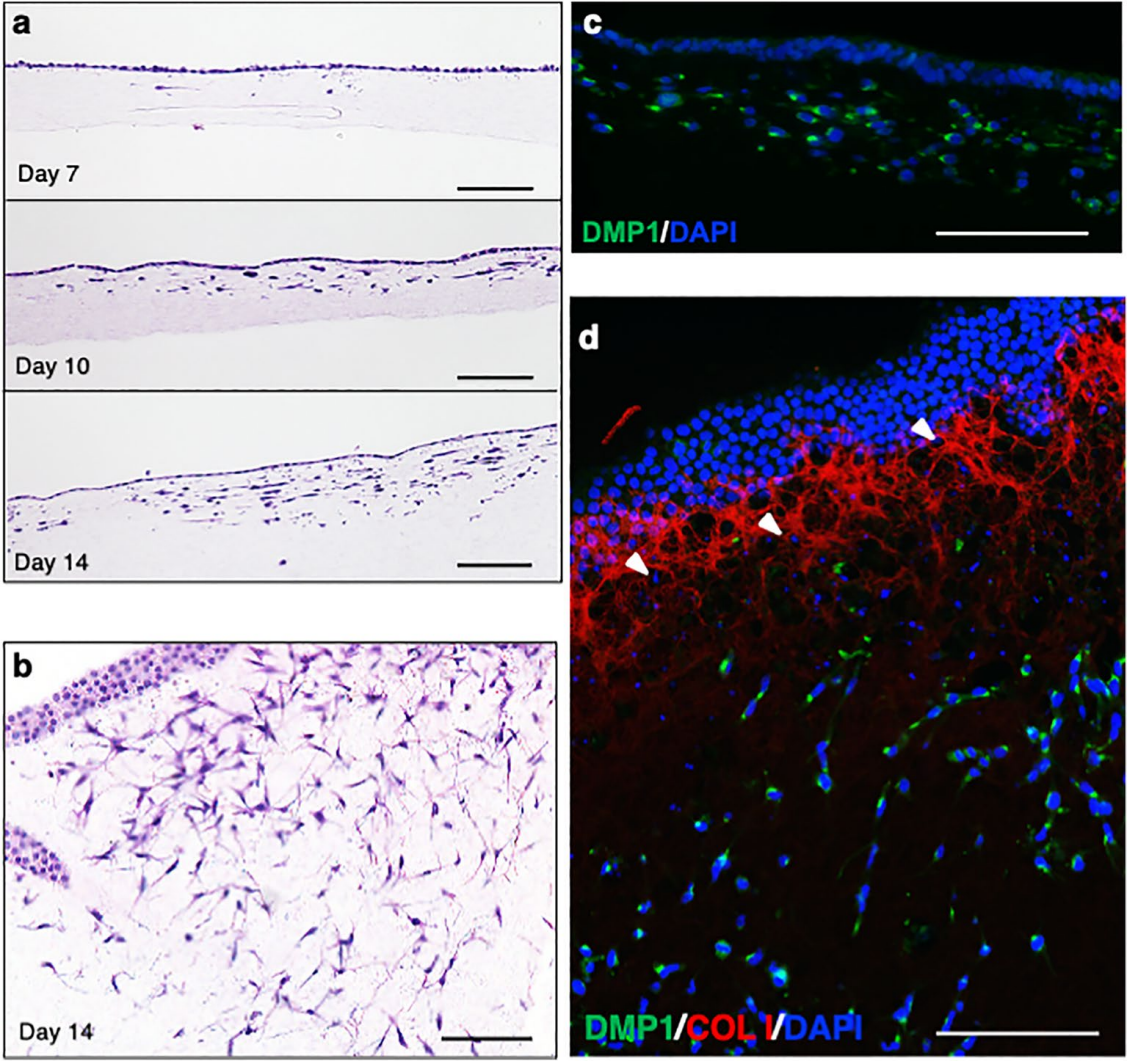
Induction of osteoblastic and osteocytic cells from hiPSCs (414C2) on type I collagen gel. (**a**) Histological findings of vertical sections of induced cells on type I collagen gel at days 7, 10 and 14 by Hematoxylin–Eosin (HE) staining. (**b**) Histological findings of a horizontal section at day 14 by HE staining. (**c**) Histological findings of a vertical section at day 14 with immunostaining for DMP1 (green) and DAPI (blue). (**d**) Histological findings of a horizontal section at day 14 with immunostaining for DMP1 (green), type I collagen (red) and DAPI (blue). Arrowheads show cells surrounded by COL I. Scale bars = 100 μm.

The osteogenic induction of hiPSCs (414C2) was performed on type I collagen gel using a previously reported method on matrigel-coated dishes^17^, and the differentiation process was observed using histological specimens. Vertical sections at each time point during the induction revealed the gradual increase of cells in the gel (Figs. 1a and S1a). Horizontal sections on day 14 demonstrated that the surface of the gel was covered by a sheet-like structure of cuboidal cells and cells inside of the gel showed a dendritic morphology connecting with adjacent cells (Figs. 1b and S1b). Immunostaining of a vertical section showed that cells inside of gels were positive for DMP1 (Fig. 1c,d) and that of a horizontal section showed a mesh-like distribution of human type I collagen (COL I) beneath the layer of cuboidal cells (Figs. 1d and S1c).

To compare the characteristics of these cells with those of established mouse cell lines, osteoblastic (MC3T3-E1) and osteocytic (MLO-Y4) cells^18^ were seeded on the collagen gel and cultured with the osteogenic induction method. In the case of MC3T3-E1, vertical sections at day 14 showed few cells in the gel (Fig. S2a), whereas many MLO-Y4 cells were found in the deep area of the gel (Fig. S2d). Horizontal sections showed multilayers of MC3T3-E1 cells on the surface (Fig. S2b), and a newly formed mesh-like Col I matrix was found beneath the cell layer (Fig. S2c). On the other hand, many MLO-Y4 cells were found in the gel (Fig. S2e), in which little Col I matrix was found (Fig. S2f.). These findings were comparable with those of osteogenic-induced hiPSCs, suggesting that the 3D culture system successfully induced osteoblastic and osteocytic cells from hiPSCs.

### Dynamic behavior of hiPSCs during osteogenic induction

**Figure 2:**
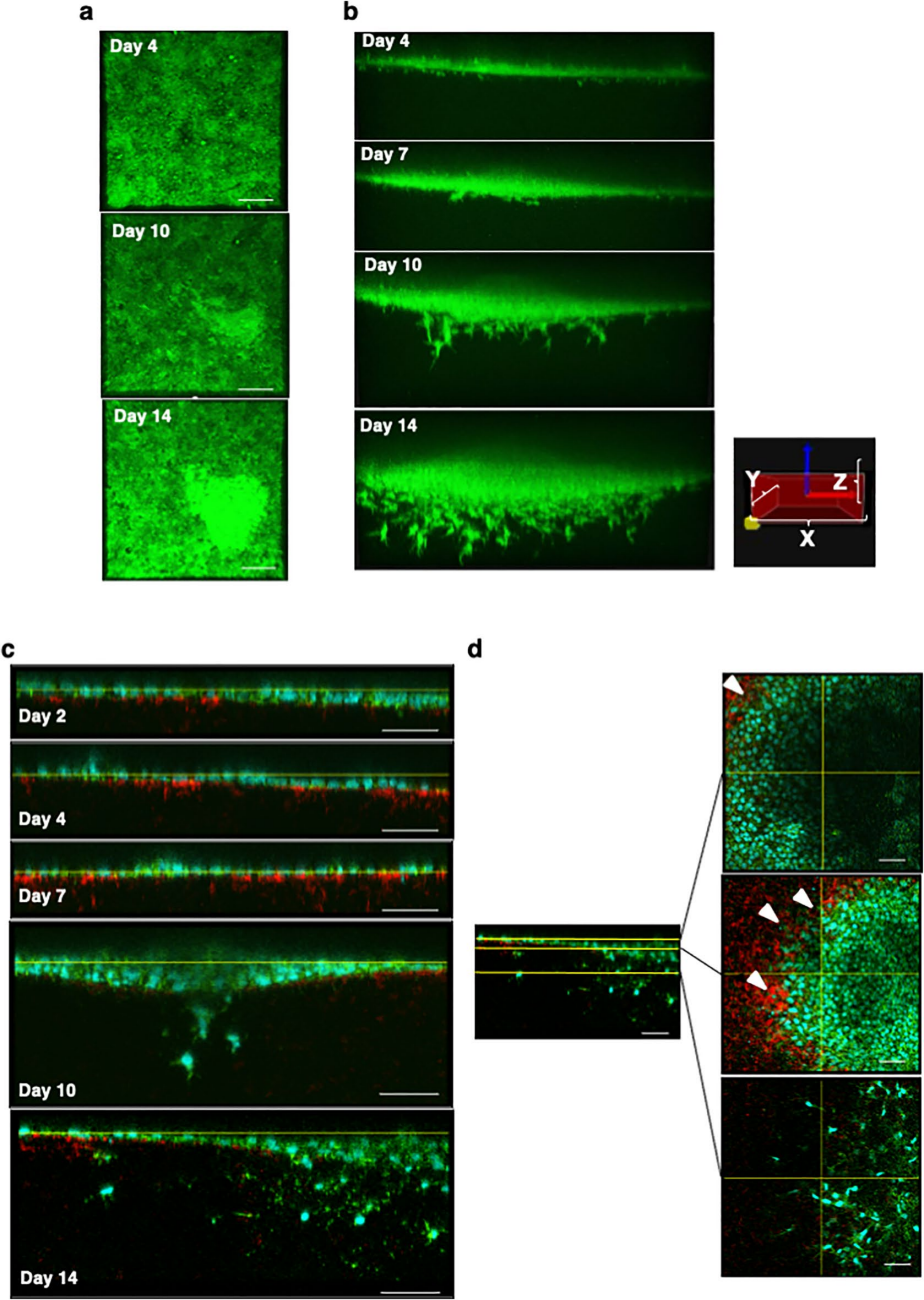
Dynamic behavior of hiPSCs during osteogenic induction. Horizontal (**a**) and vertical (**b**) snapshots of time-lapse imaging during the osteogenic induction of GFP-labeled hiPSCs (W2G) on type I collagen gel. Images were taken at the indicated times. Scale bars = 100 μm in (a) and X, Y: 508 μm and Z: 225 μm in (**b**). Confocal images of hiPSCs (404C2) in vertical (**c**) and horizontal (**d**) views during the osteogenic induction. Sections were stained with Phalloidin (green) and anti-COL 1 antibody (red). Arrowheads show cells surrounded by COL I.

As demonstrated in our previous study^17^, the dynamic behavior of hiPSCs during osteogenic induction on type I collagen gel was visualized by time-lapse imaging using GFP-labeled iPSCs. Using the same imaging, horizontal images showed that induced cells became confluent at day 4 and focal cell aggregation appeared around day 10, gradually enlarged and produced a condensed nodule at day 14 (Fig. 2a and Mov. S1). In the vertical images, cells from the aggregates beginning day 7 further invaded the deep area and developed a dendritic morphology at day 10. At day 14, the aggregated cells sank into the gel as a mass, from which many cells actively invaded the deep area and showed dendritic morphology (Figs. 2b and Mov. S2).

To investigate the process in detail, 414C2-derived cells with collagen gel were stained by Phalloidin and an antibody for COL I at different time points and analyzed by confocal microscopy. Vertical views showed COL I beneath the surface cell layer as early as day 2, and the expression of which gradually increased and mixed with pre-existing collagens from day 4 to day 7 (Figs. 2c and S3a). At day 7, focal cell proliferation began. The proliferating cells embedded into the gel as a mass at day 10, and some separated from the mass to invade the deeper area (Fig. 2c). Horizontal sections at day 14 were analyzed at several levels (Figs. 2d and S3b). The uppermost section shows a sheet-like structure of cuboidal cells, and the middle section shows cells surrounded by or burred in COL I matrix (Figs. 2d and S3b, indicated by arrowheads). The lowest section shows dendritic cells and little production of COL I. These results indicated that hiPSCs differentiated into osteoblastic and osteocytic cells through nodule formation and invasion into collagen gel.

### Location-dependent expression of stage-related markers by cells in 3D culture

**Figure 3:**
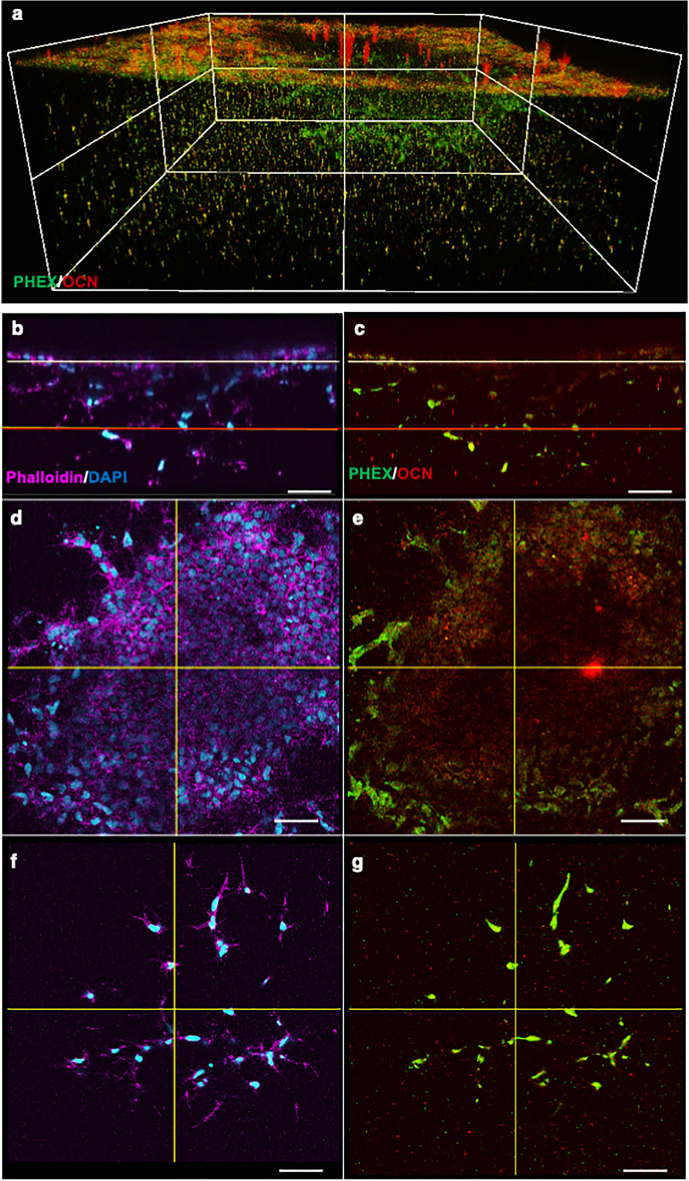
Expression of stage-related markers in 3D culture. (**a**) Reconstructed 3D view of confocal images at day 14. Collagen gels with induced cells (414C2) were stained with antibodies for PHEX (green) and OCN (red), and immunofluorescence images obtained by confocal analysis were reconstructed to build 3D images. (**b**–**g**) Immunofluorescence analysis by confocal imaging. Collagen gel with induced cells at day 14 were stained with Phalloidin (magenda) and DAPI (skyblue) (**b**,**d**,**f**) or antibodies for PHEX (green) and OCN (red) (**c**,**e**,**g**). Horizontal images were analyzed at surface (**d**,**e**) and deep (**f**,**g**) levels indicated by the white and red lines in (**b**) and (**c**), respectively. Scale bars = 50 μm.

To further characterize cells during the transition process, the expression of stage-specific markers at day 14 was visualized by immunohistochemical staining and confocal imaging. A reconstructed 3D view clearly showed differences between cells on the surface and in the gel (Fig. 3a). Cells on the surface were positive for osteocalcin (OCN), whereas cells positive for PHEX were found mostly in the gel (Fig. 3a). **Horizontal sections at different depths were further analyzed (Figs. 3b,c, and S4a). In the surface layer, cells making a sheet-like structure were positive for OCN (Fig. 3d), and some cells around nodules expressed PHEX (Figs. 3e and S4b). Cells in the deep layer showed dendritic morphology (Fig. 3f) and expressed PHEX (Figs. 3g and S4b). These sequential changes in cellular morphology and the expression of stage-specific markers indicated that the current 3D culture system recapitulates the transition of osteoblasts to osteocytes.

### Osteogenic induction of hiPSCs produced both stem cell-like cells and terminally differentiated cells

**Figure 4:**
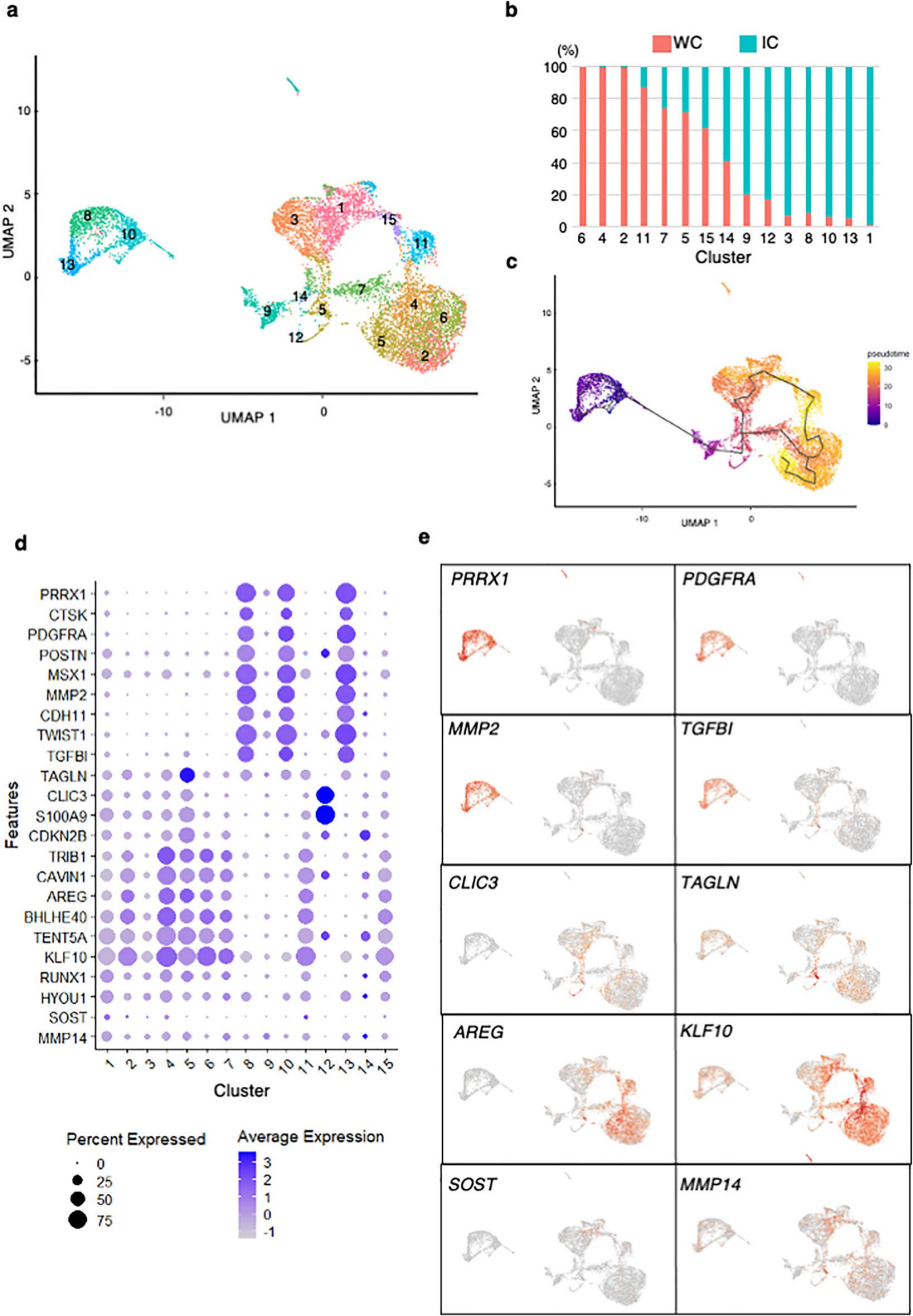
Single-cell expression profiles of osteogenic-induced cells. **(a)** UMAP plot of cell-type clusters. Cells from whole cell fraction (WC) and invading cell fraction (IC) were combined and analyzed and clustered into 15 subsets. (**b**) Relative contribution of WC- and IC-derived cells in each cluster. (**c**) Pseudotime trajectory analysis demonstrated the differentiation direction from cells colored in purple to those in yellow. (**d**) Average expression and percent expression of representative stage-related genes in each cluster with dot plots. (**e**) Visualization of cells expressing representative stage-related genes in (**d**) by UMAP.

Aggregation and invasion into the gel were observed focally, and other cells remained on the gel with a sheet-like structure, suggesting that cells were heterogenous in terms of the differentiation status even at day 14. To characterize these heterogenous cells, their expression profiles were analyzed at the single cell level. The whole cell fraction (WC) and invading cell fraction (IC) were prepared as described in the Method section and subjected to scRNA sequencing. Data obtained from WC and IC were combined and analyzed using Seurat v4, which classified the induced cells into 15 clusters, as demonstrated by UMAP (Fig. 4a) and differentially expressed genes (DEGs) in each cluster were identified as (Fig. S5). Based on the contribution of WC- and IC-derived cells in each cluster (Fig. 4b), we were able to speculate the localization of cells in some clusters. For example, clusters 2, 4 and 6 were almost exclusively composed of WC-derived cells, indicating that cells in these clusters were on the surface of the gel. On the other hand, most cells in cluster 1 were derived from IC, indicating that these cells were inside the gel. Other clusters were composed of both populations but at different ratios.

A pseudotime trajectory analysis was performed using Monocle 3 R to investigate the differentiation process and indicated cells in clusters 8, 10 and 13 were at the root of the trajectory and precursors of other cells (Fig. 4a, c). Among the differentially expressed genes (DEG) in these clusters, we identified several markers of SSCs, such as *PRRX1* and *PDGFRA* genes^19,20^ (Fig. 4d, e). Cells in these clusters also expressed *periostin* (*POSTN*) and *cathepsin K* (*CTSK*) genes (Fig. 4d), which are markers for periosteal SSCs^20,21^. These data suggested that cells in clusters 8, 10 and 13 have progenitor potential. We also found that cells in these clusters expressed several early osteoblastic-lineage marker genes regulated by the TGFβ signal, such as *cadherin 11* (*CDH11)*, *MMP2*, and *TGFBI*^22–24^(Fig. 4d).

The pseudotime trajectory analysis also indicated that cells in cluster 5 were located between SSC-like cells and differentiated cells. *Transglin* (*TAGLN*), which regulates osteoblastic differentiation from precursors through actin reorganization^25^, was identified as a DEG in this cluster (Fig. 4d,e). *CLIC3* gene was another DEG in this cluster (Fig. 4d,e) and recently identified as a lineage-specific gene regulating the differentiation of osteoblasts from MSCs^26^.

Cells in clusters 1 and 4 shared the expression of several osteoblast-related genes, although those in cluster 4 were on the surface of the gel and those in cluster 1 were inside. Some genes, such as *TRIB1*, *CAVIN1*, *amphiregulin* (*AREG)*, and *DEC1*, were preferentially expressed in the surface cluster^27–30^, whereas *KLF10* and *TENT5A* were equally expressed in both populations^31,32^. Mutations of *TENT5A* gene were recently identified in a hereditary bone disease, osteogenesis imperfecta^33^. Finally, although the number was limited, some cells in cluster 1 expressed the osteocyte marker gene *sclerostin* (*SOST)* and *MMP14*^33,34^.

These results indicated that the current induction method on type I collagen gel induced SSCs from hiPSCs, which produced differentiated cells on the surface and inside the gel.

### Cell invasion required MMP activity

**Figure 5:**
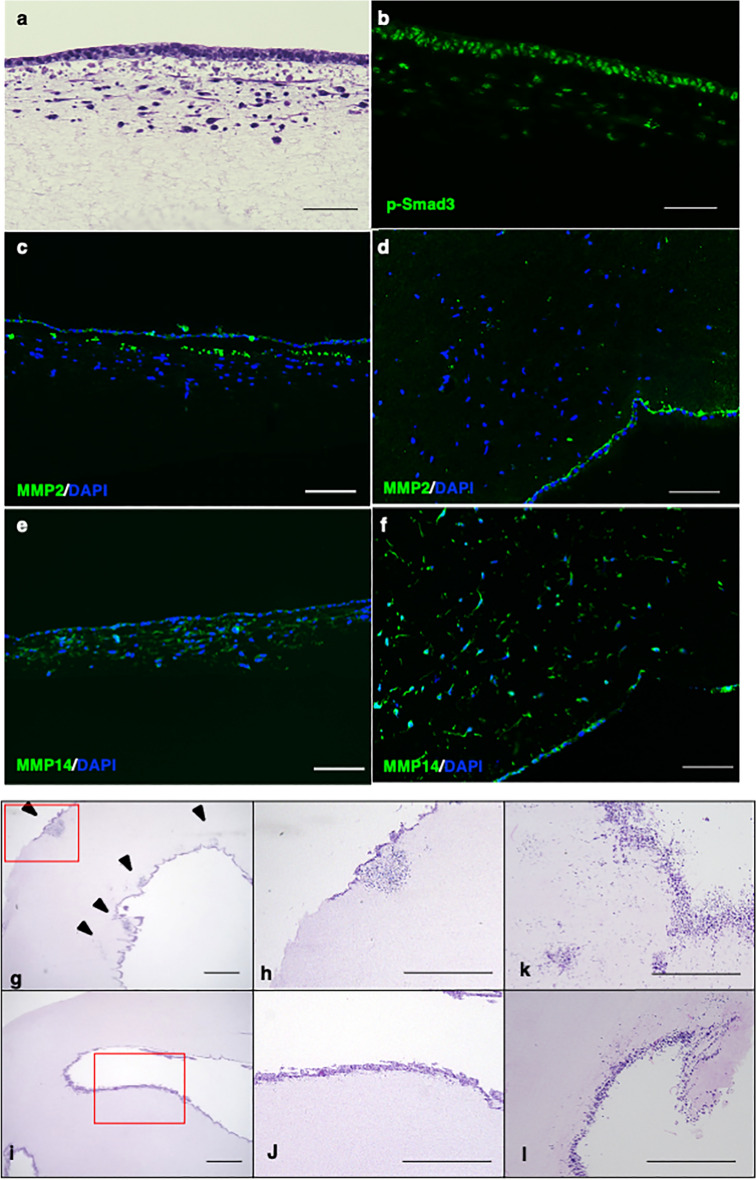
Cell invasion required the activity of MMP. Histological findings of osteogenic induced hiPSCs (414C2) on type I collagen gel at day 14. Cells cultured with collagen gel were stained with Hematoxylin–Eosin (HE) (**a**), antibodies for p-Smad3 (green) (**b**), MMP2 (green) and DAPI (blue) (**c**,**d**), or MMP14 (green) and DAPI (blue) (**e**,**f**). Images from vertical (**a**,**b**,**c**,**e**) and horizontal (**d**,**f**) sections are shown. Scale bars = 100 μm. Histological findings of osteogenically induced hiPSCs (**g**–**j**) or MLO-Y4 (**k**,**l**) at day 14 in vertical sections by HE staining. Cells were treated without (**g**,**h**,**k**) or with (**i**,**j**,**l**) GM6001 (25 μM). (**h**,**j**) are enlarged views of the red-framed areas in (**g**) and (**h**), respectively. Arrowheads in (**g**) indicate invading cells. Scale bars = 1 mm.

The results of the scRNA sequencing analysis suggested that the TGFβ signal plays a role in the maintenance of osteoblastic phenotype by regulating the expression of several genes, including *MMP2*. Vertical sections of induced cells with collagen gel at day 14 showed that phosphorylated-Smad3 was stained in cells on or just beneath the surface (Fig. 5a,b). The expression of MMP2 was also detected in these cells, but not in cells located in the deeper area of the gel (Fig. 5c,d). On the other hand, cells in the deeper area expressed MMP14 (Fig. 5e,f), which agrees with the scRNA sequencing data.

Next, a pan inhibitor of MMP, GM6001, was used to investigate the role of MMPs for cell invasion. Horizontal sections at day 14 showed the focal accumulation of cells in the gel (Fig. 5g,h), but this accumulation was almost completely inhibited by treatment with GM6001 (Fig. 5i,j). The same experiment was performed using MLO-Y4 cells, and the number of cells in the gel was clearly decreased by treatment with GM6001 (Fig. 5k,l).

### TGFβ signal regulated the morphology and motility of hiPSC-derived osteogenic cells

**Figure 6:**
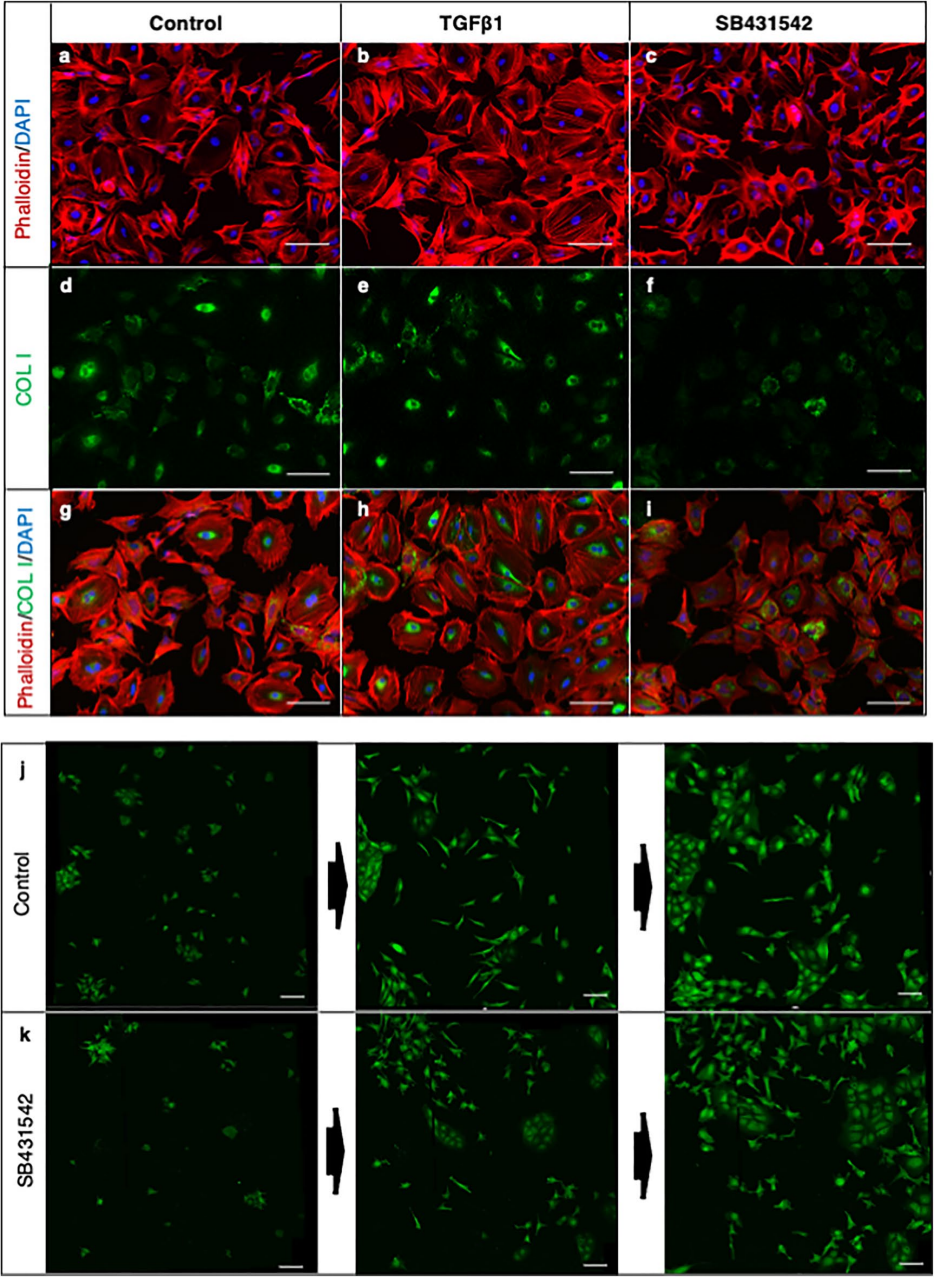
Effect of TGFβ signal on osteogenic-induced iPSCs on type I collagen gel. The whole cell fraction of osteogenic-induced hiPSCs (414C2) was isolated at day 14 and cultured for 48 h in control medium (**a**,**d**,**g**) or medium supplemented with TGFβ1 (5 ng/mL) (**b**,**e**,**h**) or SB431542 (10 µM) (**c**,**f**,**i**). Cells were stained with Phalloidin (red) and DAPI (blue) (**a**–**c**) or antibody for COL I (**d**–**f**). (**g**–**i**) Merged images. Snapshots of the time-lapse imaging of isolated cells from days 1 to 3 cultured in control medium (**j**) or medium supplemented with SB431542 (10 µM) (**k**). Scale bars = 100 μm.

Finally, we evaluated the effect of the TGFβ signal on cells isolated from 3D culture at day 14. Cells in WC showed heterogenous cell morphology, including osteoblast-like and osteocyte-like cells (Fig. 6a), and osteoblast-like cells vigorously proliferated again and produced a sheet-like structure, among which osteocytic cells rapidly migrated but rarely divided (Mov. S3). Cuboidal osteoblastic cells became dominant after treatment with TGFβ1 (Fig. 6b), whereas treatment with a TGFβ signal inhibitor (SB431542) increased cells with dendritic, osteocytic morphology (Fig. 6c). Immunostaining of COL I showed reduced COL 1 production upon inhibition of the TGFβ signal (Fig. 6d–i). Time-lapse imaging showed different cell behaviors. In control medium, cuboidal cells vigorously proliferated, making a sheet-like structure, and some dendritic cells became cuboidal (Fig. 6j and Mov. S4). On the other hand, SB431542 treatment reduced the proliferation of the cuboidal cells and enhanced the movement of dendritic cells (Fig. 6k and Mov. S5).

These effects of the TGFβ signal on cellular behavior were also observed in experiments using MLO-Y4 cells. MLO-Y4 is an osteocytic cell line, but the morphology under standard culture condition is heterogenous, showing both polygonal osteoblastic and dendritic osteocytic morphologies (Fig. S6a). Under culture with TGFβ1, cells with polygonal morphology became dominant (Fig. S6b), whereas cells with dendritic morphology became dominant under culture with SB431542 (Fig. S6c).

These data indicated that the TGFβ signal maintains the osteoblastic phenotype of osteogenic-induced hiPSCs, and therefore reduction of the signal is required for the transition process from osteoblasts to osteocytes.

## Discussion

Here we show using hiPSCs and mouse cell lines that the TGFβ signal maintains the osteoblastic phenotype and its downregulation is critical for the differentiation to osteocytes. This realization came from a new model that uses 3D culture gels and a rapid differentiation method that includes retinoic acid. The biological features of osteoblasts and osteocytes have been previously investigated using primary isolated cells and established cell lines. Primary isolated cells show extensive heterogeneity in terms of gene expressions and biological functions, and it is difficult to continue to culture them due to their low proliferative activity. Additionally, SV40 large T has been used to establish immortalized cell lines^18^, but, although useful, these cells may have unnatural phenotypes due to oncogenic effects of large T antigen.

Recent progress in stem cell biology has enabled the use of PSCs including iPSCs as a cellular source to obtain osteogenic cells by appropriate step-wise induction methods for each of neuroectoderm, paraxial mesoderm, and lateral plate mesoderm lineages^3–5^. In each developmental route, the robustness of the induction is confirmed by the expression of markers specific for intermediate cells such as neural crest derived mesenchyme, sclerotome, and limb bud mesoderm^3–5^. The final step is common in all routes: the induction of mineralized nodules by osteogenic medium in 2D culture, in which dense culture conditions favor the osteogenic commitment of precursor cells^35^. From the condensation of cultured cells, bone-like nodule formation is observed during the induction^36–38^. In the present study, the high proliferative capacity of hiPSCs was exploited to make a condensed cell aggregate within seven days, which led to the rapid induction of osteogenic cells. Furthermore, the 3D culture condition may facilitate the differentiation, as demonstrated with MC3T3-E1^39^.

The induction of osteocytes, however, is difficult in most cases. One reason is because cell–matrix interactions are involved in the transition process from osteoblasts to osteocytes but are difficult to recapitulate in vitro. We used a 3D culture system to mimic the physiological circumference, making the dynamic observation of the whole process of migration possible by time-lapse imaging with GFP-labeled hiPSCs. Osteoblast-like cells on the surface become multi-layered (condensation), and some were trapped in self-produced matrix and actively migrated into the deep layer of the collagen gel. These findings supported the reported hypothesis that the initial step of burying is likely due to passive entrapment, but migration into the deeper layer is caused by active movement^40^. Three mechanisms have been proposed for the interaction between type I collagen and osteocytes, and the current system showed that motile cells moved into the collagen network. Future studies using cells labeled with osteocyte markers, such as DMP1^41,42^, and our induction method will provide more information about the interaction.

To investigate the differentiation process from hiPSCs to osteocytes using the current induction method, scRNA sequencing was performed^43–46^. One common finding from the scRNA sequencing of primary bone tissues is the presence of stem cell populations. The scRNA sequencing of neonatal mice calvariae tissues identified a precursor fraction characterized by several genes including *Mfap4*^43^. The same study showed that endocortical cells recovered from collagenase-treated long bone tissues contained three types of mesenchymal-lineage cells represented by the expression of *Pdgfra*, *Acta2*, and *Cxcl12*^43^. The scRNA sequencing of Col1-labeled cells isolated from newborn mouse calvariae defined several clusters that showed different stages of differentiation^44^. Even among cells in the same cluster, the expression profiles were highly heterogeneous. One interesting finding in that report is that cell clusters with stem cell markers, such as *Nestin* (*Nes*) and *Nidogen 1*(*Nid1*), were developed from differentiated cells, suggesting the plasticity of osteogenic cells, which may retain or reacquire progenitor properties^44^.

The current study is the first to analyze the osteogenic differentiation process of hiPSCs by scRNA sequencing, which identified multiple clusters including those with features of SSCs. SSCs are a relatively new concept derived from MSCs, which are considered to possess at least tri-lineage differentiation potential^1,8^. Recent analyses including those using scRNA sequencing data have identified two SSC subgroups, periosteal SSCs and bone marrow or perivascular SSCs^6^. The former is an osteochondral progenitor cell type without adipogenic potential, and the latter is a true tri-lineage stem cell type. Although we were unable to analyze the differentiation potential of SSC-like cells in clusters 8, 10, and 13 here, their expression profile, which included *PRRX1*, *CTSK*, and *POSTN* genes, was consistent with reports for periosteal SSCs^19–21^. Interestingly, cells in these clusters also expressed several genes identified in stem cell populations in previous studies such as *Mfap4, Pdgfra, Nes* and *Nid1* genes^20,43,44^ (Figs. 4d,e, and S7).

One feature of the stem cell population in the current study was the up-regulation of several TGFβ-regulated genes, including *TGFBI*, *CDH11*, and *MMP2*, all of which are involved in the cell adhesion process. TGFBI encodes an RGD-containing protein that binds to type I collagens and plays a role in cell-collagen interactions^24^. CDH11, also known as OB-cadherin, is a member of the cadherin superfamily and mediates calcium-dependent cell–cell adhesion^23^. A recent study using artificial matrix components showed that the production of active MMP2 by SSCs is an important factor for osteogenesis by providing high levels of intracellular tension^47^. Although TGF-regulated genes in other lineages such as *COL2A1* in the chondrogenic lineages was positive in some cells in these clusters (data not shown) and therefore not all cells in these clusters were committed to the osteogenic lineage, these observations indicate the important role of the TGFβ signal for maintaining the status of SSCs and immature osteoblasts. In other words, down-regulation of the TGFβ signal is required for further differentiation to mature osteoblasts and osteocytes. TGFβ promotes osteogenesis by recruiting progenitor cells but inhibits late differentiation, indicating it has biphasic effects^48,49^. Modification of the TGFβ signal in isolated cells agreed with this concept (Figs. 6 and S7). Cells treated with TGFB1 showed a polygonal feature with stretched actin fibers and maintained cell–cell adhesion to make a sheet-like structure, whereas those treated with a TGFβ signal inhibitor showed dendritic features and vigorous motility. In a spheroid culture of MSCs, the inhibition of F-actin polymerization accelerated the differentiation of osteocytes^12^, suggesting the possibility that actin depolymerization is necessary for osteocytogenesis. Identification of critical genes in each step from DEGs in each cluster will be a future issue to be done.

In conclusion, we established a 3D culture system to analyze and visualize the process of osteogenic differentiation from hiPSCs to osteocytes and found the TGFβ signal has an essential role for the osteoblast-to-osteocyte transition. Future studies using gene-modified hiPSCs in combination with our system may provide a platform to analyze the role of each factor in osteogenic differentiation and also serve as a tool to identify therapeutic drugs for intractable bone diseases.

## Experimental Procedures

### iPSCs and cell culture

Standard hiPSCs 414C2 and 409B2 were used as reported previously^50^. GFP-labeled hiPSCs were prepared from 409B2 to include constitutively active GFP in on-feeder culture condition^51^. On-feeder hiPSCs were maintained in primate embryonic stem cell medium (ReproCELL) supplemented with 4 ng/mL recombinant human basic fibroblast growth factor (FGF2; Wako) and penicillin–streptomycin (Thermo). Three days before the induction of osteogenic-lineage, on-feeder hiPSCs were transferred to a Matrigel-coated dish and cultured in mTeSR1 medium (STEMCELL Technology).

### Osteogenic induction of hiPSCs

The osteogenic induction of hiPSCs using a previously reported method^17^ was performed on a dish or plate coated with porcine type I collagen gel (Cellmatrix, Nitta gelatin). In brief, 1.5 X 10^6^ or 6 X 10^5^ hiPSCs were seeded in a 3.5-cm glass bottom dish (for confocal imaging) or a 12-well plate (for RNA extraction and tissue sectioning) coated by collagen gel and cultured with a mixed medium of mTeSR1 (20%) and osteogenic induction (OI) medium (80%), which was composed of KnockOut™ DMEM (Thermo) supplemented with 20% FBS (Nichirei), 2 mM Gluta-MAX ™ (Thermo), 10 mM glycerol-2-phosphate (Sigma), 1 nM dexamethasone (Sigma), 0.1 mM 2-mercaptoethanol (Thermo), 50 μg/mL L-ascorbic acid 2-phosphate sesquimagnesium salt hydrate (Nacalai Tesque), and 1% non-essential amino acids (Thermo) in the presence of Y-27632 (10 μM, Wako) and 1 μM retinoic acid (Wako). On day 2, the mixed medium was replaced with 100% OI medium and refreshed at days 4, 7, 9, and 11.

### Osteogenic induction of established cell lines

For the induction of 3D osteogenic induction from MC3T3-E1 and MLO-Y4, 1.5 X 10^6^ or 6 X 10^5^ hiPSCs were seeded in a 3.5-cm glass bottom dish (for confocal imaging) or a 12-well plate (for RNA extraction and tissue sectioning) coated by collagen gel, and cultured with osteogenic induction medium, which was composed of α-MEM (Gibco) supplemented with 10% FBS (Hyclone), 0.1 mM dexamethasone (Sigma), 50 mg/mL ascorbic acid (Nacalai Tesque), and 10 mM β-glycerophosphate (Sigma)) and the medium was refreshed at day 2, 4, 7, 9, and 11.

### Preparation of single cells from 3D culture

To prepare induced cells for scRNA sequencing, cultured cells on collagen gel were washed twice in phosphate-buffered saline (PBS). To obtain whole cell populations (whole cell fraction; WC), cells with collagen gel were harvested by scraping and transferred to a 5-mL tube and treated with 0.1% collagenase (Nitta gelatin) for 60 min at 37℃ with shaking. Then the cells were treated with TrypLE (Thermo) for 5 min at 37℃. After pipetting about 6 times, the cell suspension was filtrated to remove doublets. To obtain cell populations without cells on the surface of the gel (invading cell fraction; IC), cells with collagen gel were harvested by scraping and transferred to a 5-mL tube and treated with 0.1% collagenase (Nitta gelatin) for 20 min at 37℃. Then the cell suspension was filtrated to remove cells forming a sheet-like structure and further treated with TrypLE for 5 min at 37℃ twice. Finally, the cell suspension was filtrated again to remove doublets.

### Immunofluorescence analysis in 3D culture

Collagen gels with induced cells were fixed with PBS containing 4% paraformaldehyde for 60 min at 4℃, permeabilized by 0.1% TritonX-100-PBS, blocked with 3% FBS (Hyclone) for 60 min at 4℃ and incubated overnight at 4℃ with primary antibodies diluted in PBS. The samples were washed 3 times with 0.1% BSA in PBS and incubated overnight at 4℃ with secondary antibodies diluted in PBS. Nuclei were stained with 1:5000 DAPI (Sigma). The samples were observed using a FLUOVIEW FV3000 (Olympus) and analyzed using FV31S-SW software (Olympus). The antibodies used are listed in Table S1c.

### Immunofluorescence analysis in 2D culture

Cells were fixed with PBS containing 2% paraformaldehyde for 20 min at 4℃, permeabilized by 0.1% TritonX-100-PBS, blocked with Blocking One (Nacalai Tesque) for 60 min at 4℃ and incubated overnight at 4℃ with primary antibodies diluted in 10% (v/v) Blocking One in PBS-T (PBS with 0.2% Triton X-100 solution (Nacalai Tesque)). The samples were washed 3 times with PBS-T and incubated for 1 h at room temperature with secondary antibodies diluted in 10% Blocking One in PBS-T. Nuclei were stained with 1:5000 DAPI (Sigma). The samples were observed with a BZ-9000E (KEYENCE CORPORATION). The antibodies used are listed in Table S2c.

### Time-lapse imaging of 3D culture

3D live imaging was taken by a specially assembled inverted multiphoton microscope combined with a full-sized CO_2_/O_2_ incubator (LCV-MPE)^52^. The stage incubator was adjusted to the cell culture condition of 37 ℃, 95% humidity and 5% CO_2_. Scan settings were identically set for all experiments, and illumination was constant throughout the whole time period. Images were 512 × 512 pixels in resolution and taken at 1-µm depth in the z-direction (226 µm in total) at a 90-min time interval for a total duration of 7 or 8 days (from days 4 to 12 for the surface imaging and days 7 to 14 for the 3D imaging after seeding the cells). Videos were visualized in 3D using FV31S-SW.

### Time-lapse imaging of 2D culture

Cell culture plates were placed on STRG-WELSX-SET (Tokaihit) equipped with an incubation chamber and motorized stage. The stage incubator was adjusted to the cell culture condition of 37℃, 95% humidity and 5% CO_2_. Images were taken by a confocal laser scanning microscope (FV3000), scan settings were identically set for all experiments, and illumination was constant throughout the whole time period. Images were 512 × 512 pixels in resolution and taken at a 30-min time interval for a total duration of 48 h after medium change with or without SB431542. Videos were visualized in 3D using FV31S-SW. 64 regions (8 × 8) were scanned in Fig. S3, and 36 regions (6 × 6) were scanned in Figs. S4 and S5.

### Single-cell RNA sequencing

Single cell suspensions were prepared from the WC and IC using one and six wells of a 6-well plate, respectively. Each suspension was loaded on a Chromium Single Cell B Chip, and libraries of scRNA sequencing were generated on Chromium controller using the Chromium Single Cell 3′ Library & Gel Bead Kit v3 (10 × Genomics) according to the manufacturer's protocol. Sequencing libraries were loaded on an Illumina HiSeq2500 using the following read lengths: 98 bp Read1, 8 bp i7 Index, and 26 bp Read2. The conversion of BCL files to FASTQ files was conducted using bclfastq 2.20.0.422. Cell Ranger count was applied to each FASTQ file to produce a feature barcoding and gene expression library using the GRCh38 reference genome available at the Cell Ranger website. Clustering analysis was performed using Seurat R package v4.1.0. Differential expression analysis was performed using the Loupe Browser. Dot plots and violin plots were generated using the ggplot2 package in R. Monocle 3 R package v1.0.0 was used for the pseudotime analysis.

### Study approval

The experimental protocols dealing with human cell lines were approved by the Ethics Committee of the Department of Medicine and Graduate School of Medicine, Kyoto University.

### Statistical analysis

No pre-specified effect size was calculated, and no statistical method was used to predetermine the sample size. All results are biological replicas from three independent experiments and presented as means ± s.e.m. *P* values were calculated using the two-sided Student`s t-test or Dunnett’s multiple comparison test, and *P* values less than 0.05 were considered significant.

## Supplementary Information

Supplementary Information 1.

Supplementary Information 2.

Supplementary Information 3.

## Data Availability

scRNA sequencing data were deposited in the NCBI’s Gene Expression Omnibus (GEO) database (GSE186403).

## Abbreviations

GFP: Green fluorescent protein
TGFβ: Transforming growth factor beta
MMP: Matrix metalloproteinase
DMP1: Dentin Matrix Acidic Phosphoprotein 1
PHEX: Phosphate regulating endopeptidase homolog X-linked
UMAP: Uniform manifold approximation and projection
PRRX1: Paired Related Homeobox 1
PDGFRA: Platelet Derived Growth Factor Receptor Alpha
TGFBI: Transforming Growth Factor Beta Induced
CLIC3: Chloride Intracellular Channel 3
TRIB1: Tribbles Pseudokinase 1
CAVIN1: Caveolae Associated Protein 1
DEC1: Differentially Expressed In Chondrocytes 1
KLF10: Kruppel Like Factor 10
TENT5A: Terminal Nucleotidyltransferase 5A
Mfap4: Microfibril Associated Protein 4
Acta2: Actin Alpha 2, Smooth Muscle
Cxcl12: C-X-C Motif Chemokine Ligand 12
RGD: Arg-Gly-Asp
