# Supplementary material for: 3D osteogenic differentiation of human iPSCs reveals the role of TGFβ signal in the transition from progenitors to osteoblasts and osteoblasts to osteocytes
Source: Sci Rep. 2023 Jan 19;13:1094. doi: 10.1038/s41598-023-27556-w (PMC9852429; doi:10.1038/s41598-023-27556-w)
Supplement: Supplementary file 1 — Supplementary Information 1. [file 41598_2023_27556_MOESM1_ESM.pdf]

Legends for Supplementary Movies

Movie S1. Time-lapse movie of GFP-labeled hiPSCs during osteogenic induction on type I collagen gel from days 4 to 12.

Movie S2. Time-lapse movie of GFP-labeled hiPSCs during osteogenic induction on collagen gel from days 7 to 14 with 3D reconstruction.

Movie S3. Time-lapse movie of osteogenic-induced GFP-labeled hiPSCs isolated from collagen gel at day 14 and then cultured for 3 days.

Movie S4. Time-lapse movie of GFP-labeled hiPSCs isolated from collagen gel at day 14 and then cultured for 3 days in control medium after seeding.

Movie S5. Time-lapse movie of GFP-labeled hiPSCs isolated from collagen gel at day 14 and then cultured for 3 days in medium with SB431542 after seeding.
