# Supplementary figures and images for: 3D osteogenic differentiation of human iPSCs reveals the role of TGFβ signal in the transition from progenitors to osteoblasts and osteoblasts to osteocytes
Source: Sci Rep. 2023 Jan 19;13:1094. doi: 10.1038/s41598-023-27556-w (PMC9852429; doi:10.1038/s41598-023-27556-w)

## Slide 1
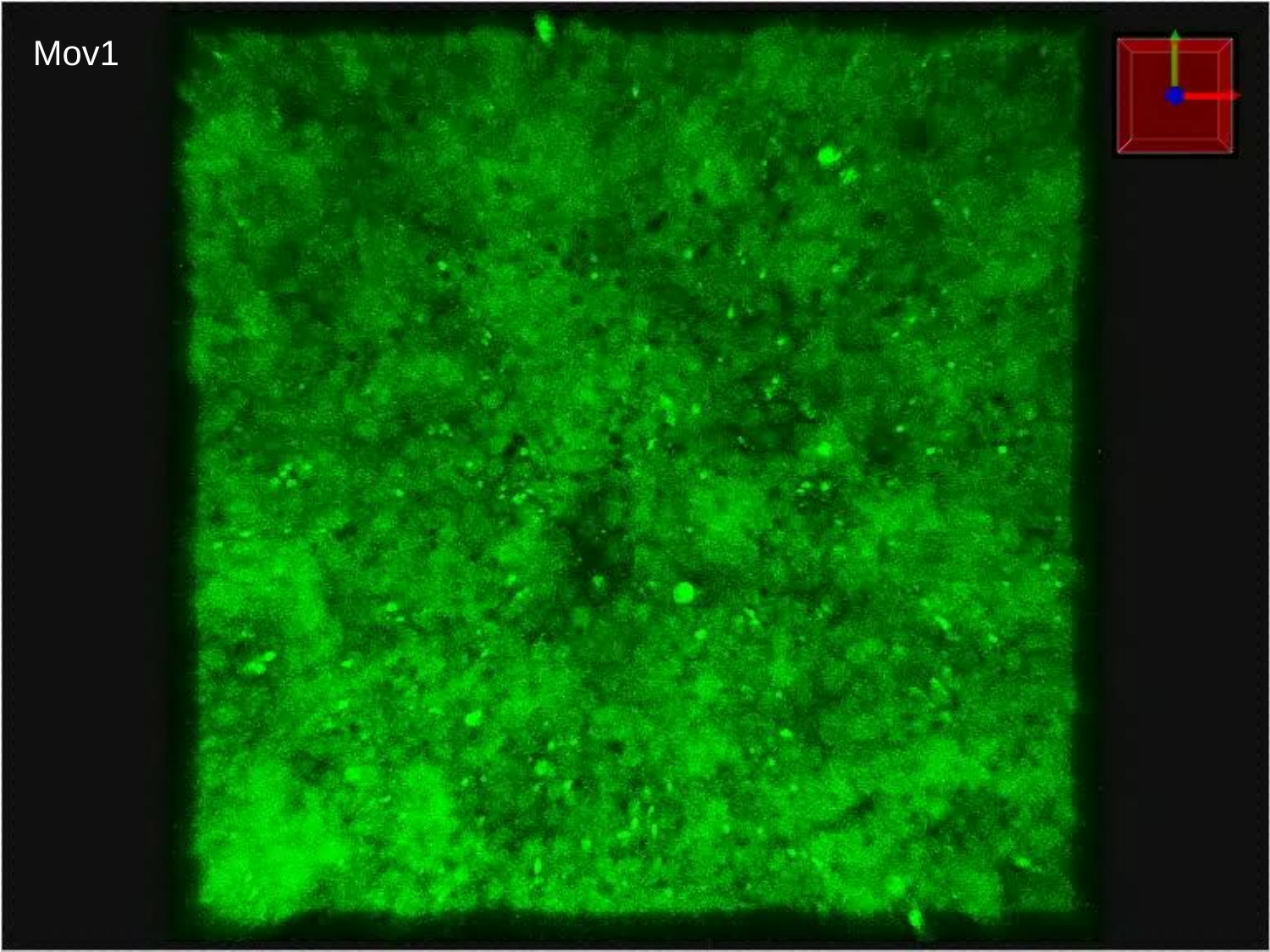

Mov1

## Slide 2
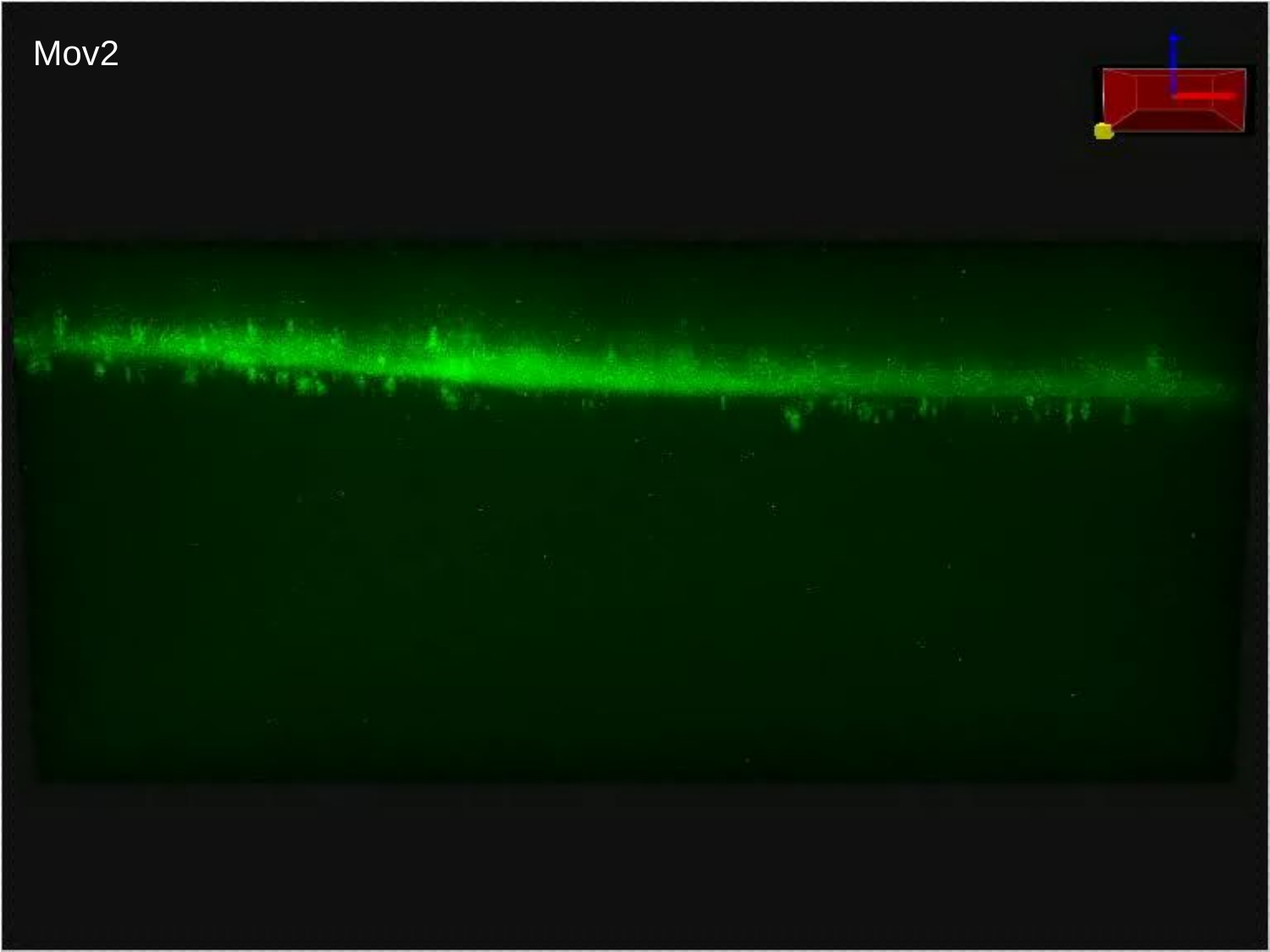

Mov2

## Slide 3
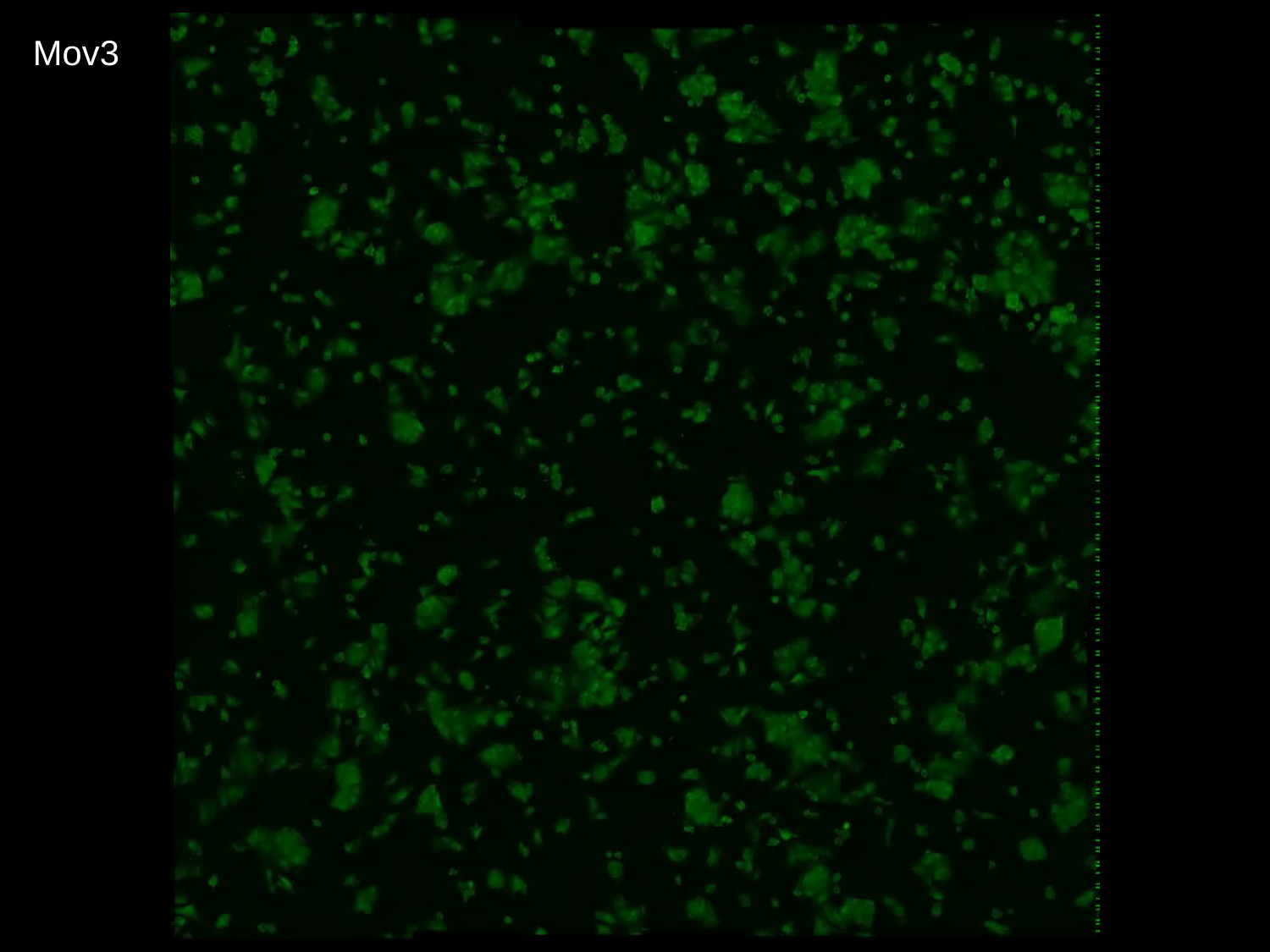

Mov3

## Slide 4
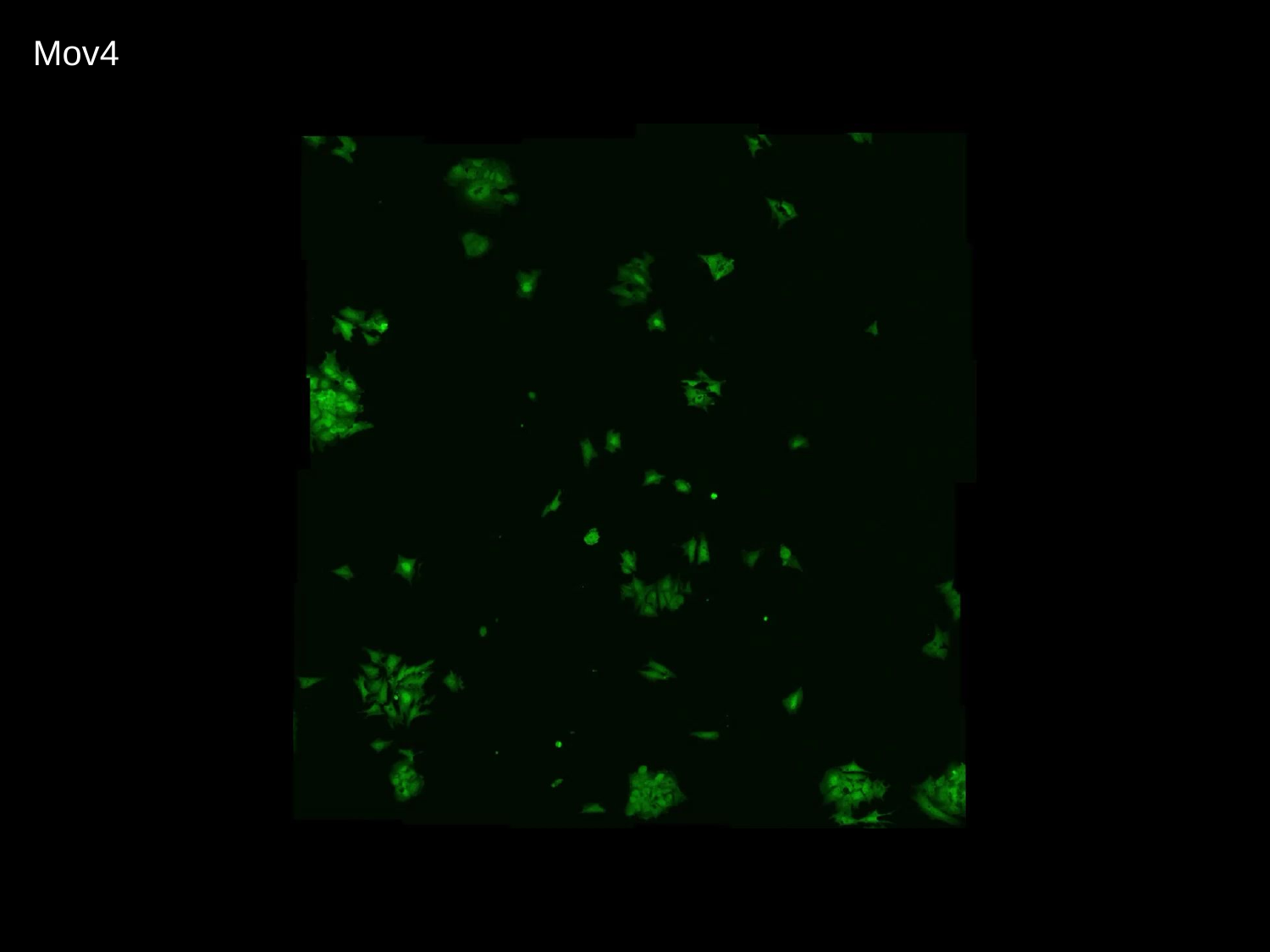

Mov4

## Slide 5
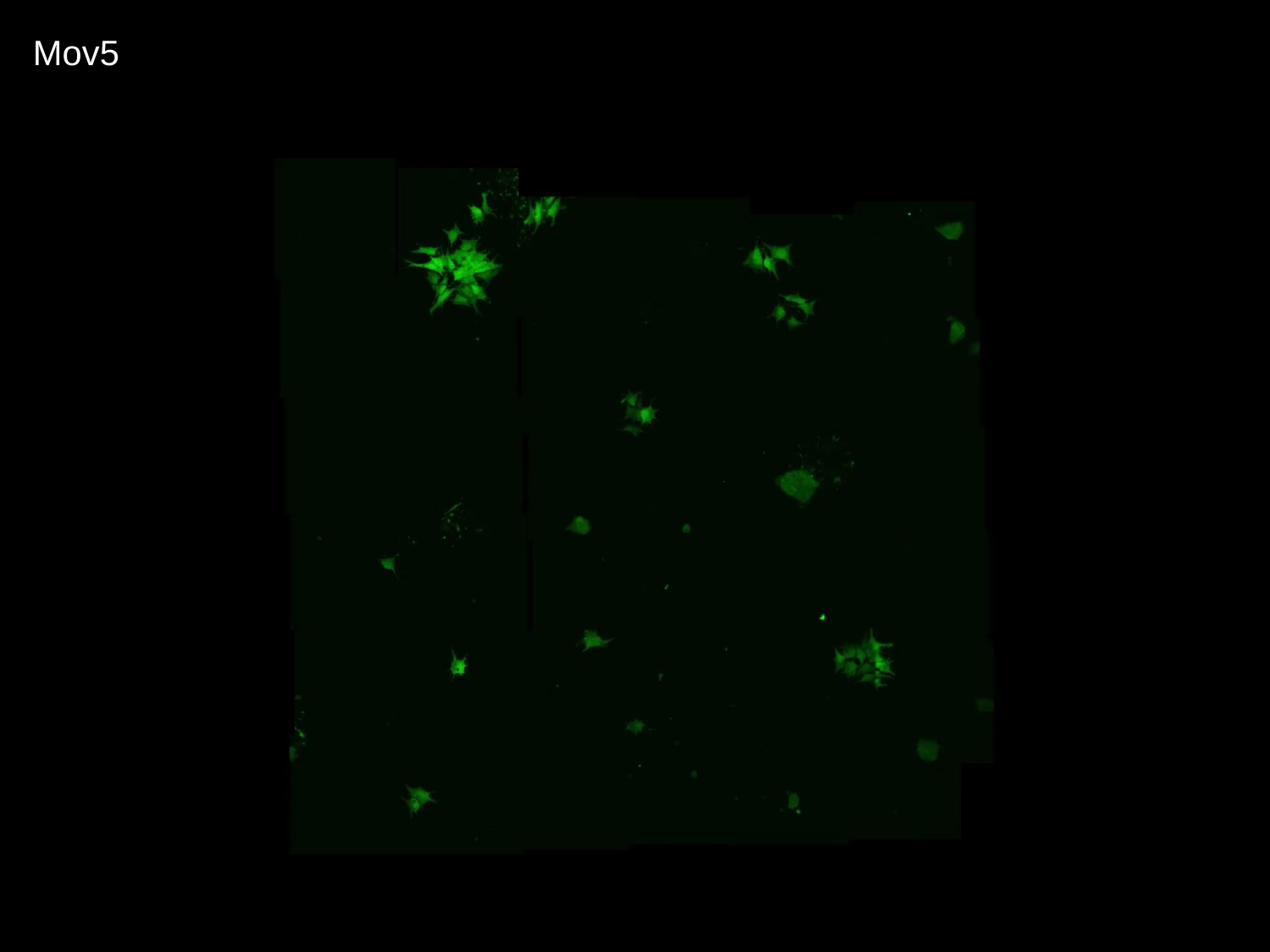

Mov5

Supplement: Supplementary file 2 — Supplementary Information 2. [file 41598_2023_27556_MOESM2_ESM.pptx]
