# Supplementary material for: 3D osteogenic differentiation of human iPSCs reveals the role of TGFβ signal in the transition from progenitors to osteoblasts and osteoblasts to osteocytes
Source: Sci Rep. 2023 Jan 19;13:1094. doi: 10.1038/s41598-023-27556-w (PMC9852429; doi:10.1038/s41598-023-27556-w)
Supplement: Supplementary file 3 — Supplementary Information 3. [file 41598_2023_27556_MOESM3_ESM.pdf]

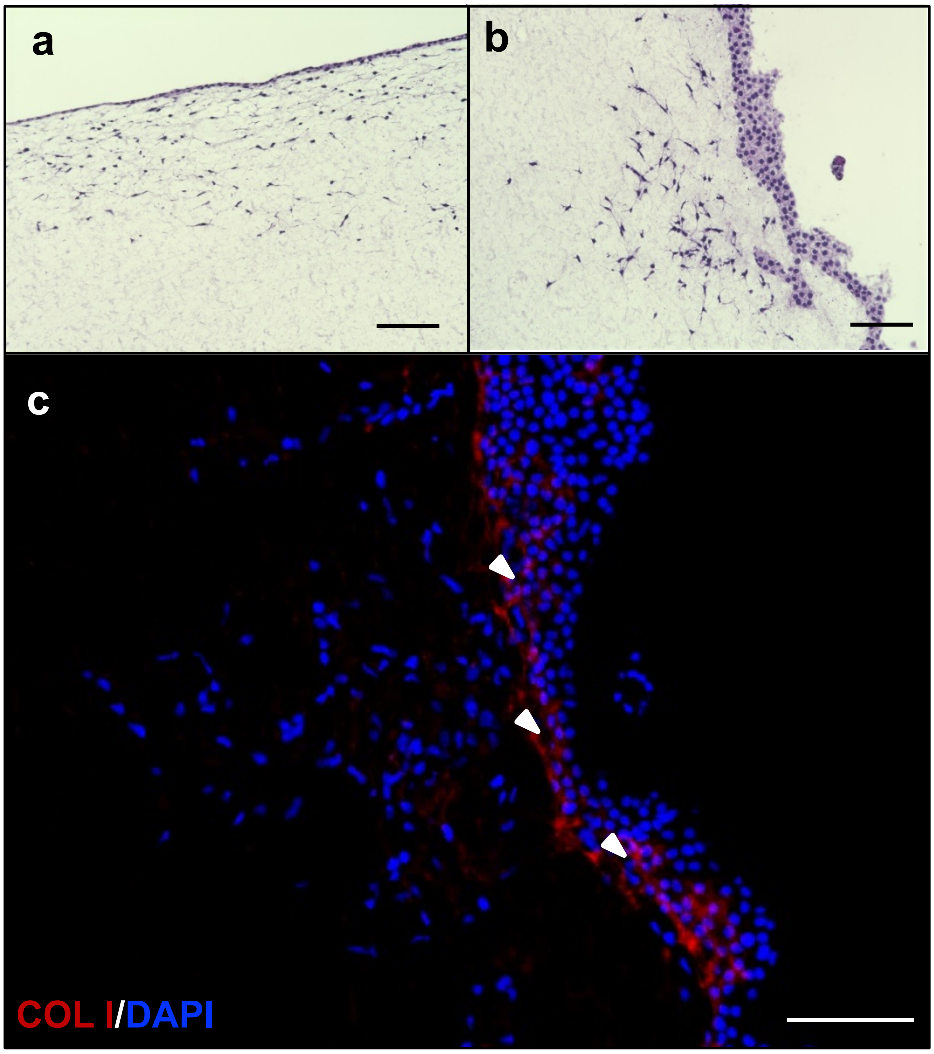

Figure S1. Induction of osteoblastic and osteocytic cells from hiPSCs (409B2) on type I collagen gel. Histological findings of vertical (**a**) and horizontal (**b**) sections of induced cells on type I collagen gel at day 14 by HE staining. (**c**) Histological findings of a horizontal section of induced cells on type I collagen gel at day 14 by immunostaining with COL I (red) and DAPI (blue). Arrowheads show cells surrounded by COL I. Scale bars = 100 μm.

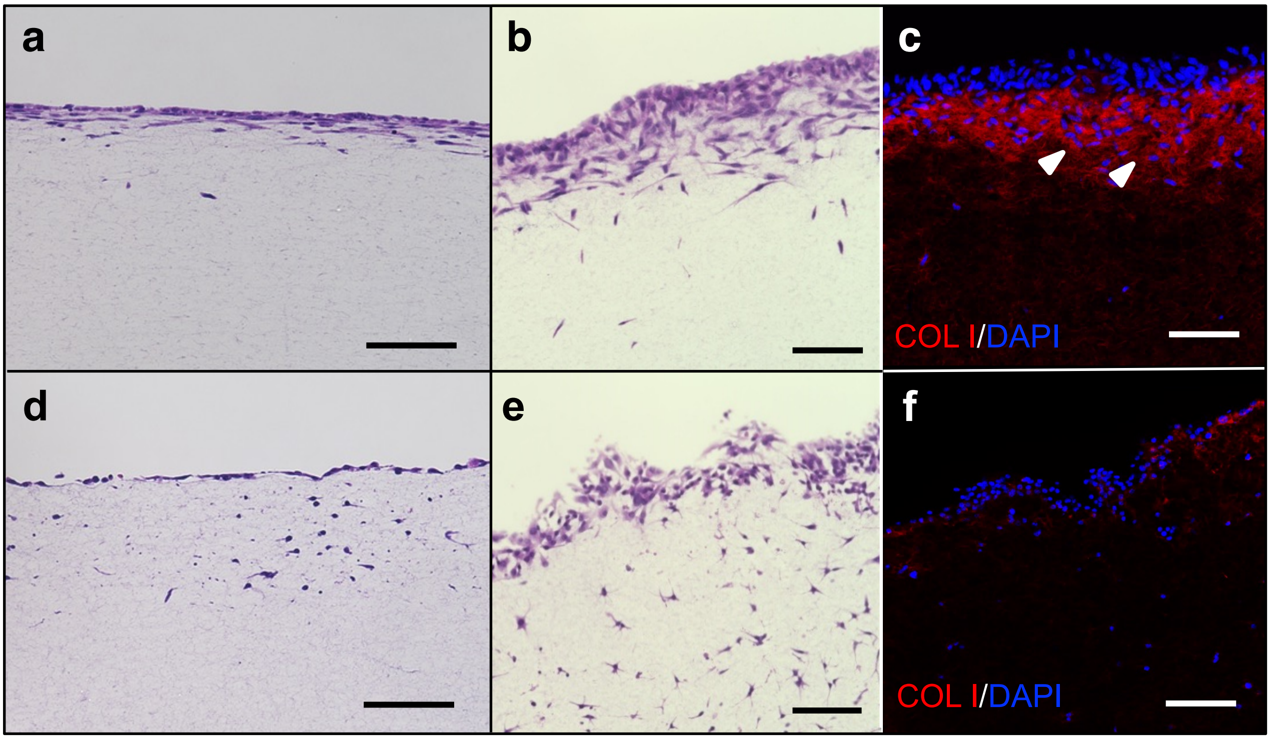

Figure S2. Osteogenic induction of osteoblastic and osteocytic cell lines on type I collagen gel. Histological findings of vertical (**a** and **d**) and horizontal (**b, c, e and f**) sections of MC3T3-E1 (**a**-**c**) and MLO-Y4 (**d**-**f**). Cells with gel were stained with HE (**a**, **b**, **d** and **e**), or DAPI (blue) and anti-COL I antibody (red) (**c** and **f**). Arrowheads indicate buried cells in COL I matrix. Scale bars = 100 µm.

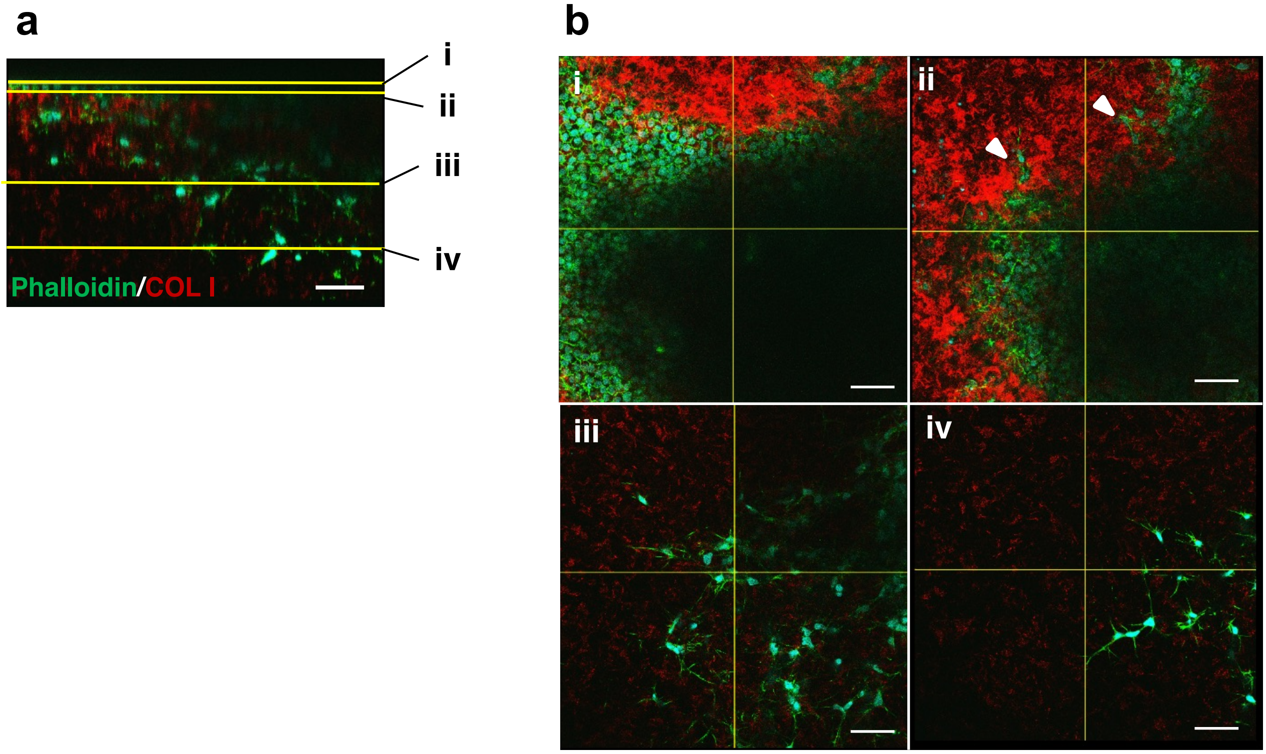

Figure S3. Confocal imaging of osteogenic-induced hiPSCs (409B2) in 3D culture – Phalloidin and COL I. Immunofluorescence analysis by confocal imaging of vertical (**a**) and horizontal sections (**b**) of 3D culture at day 14. Roman numerals in the horizontal sections indicate the level taken from the vertical sections. Cells cultured with gel were stained with Phalloidin (green) and a COL I antibody (red). Scale bars = 50 µm.

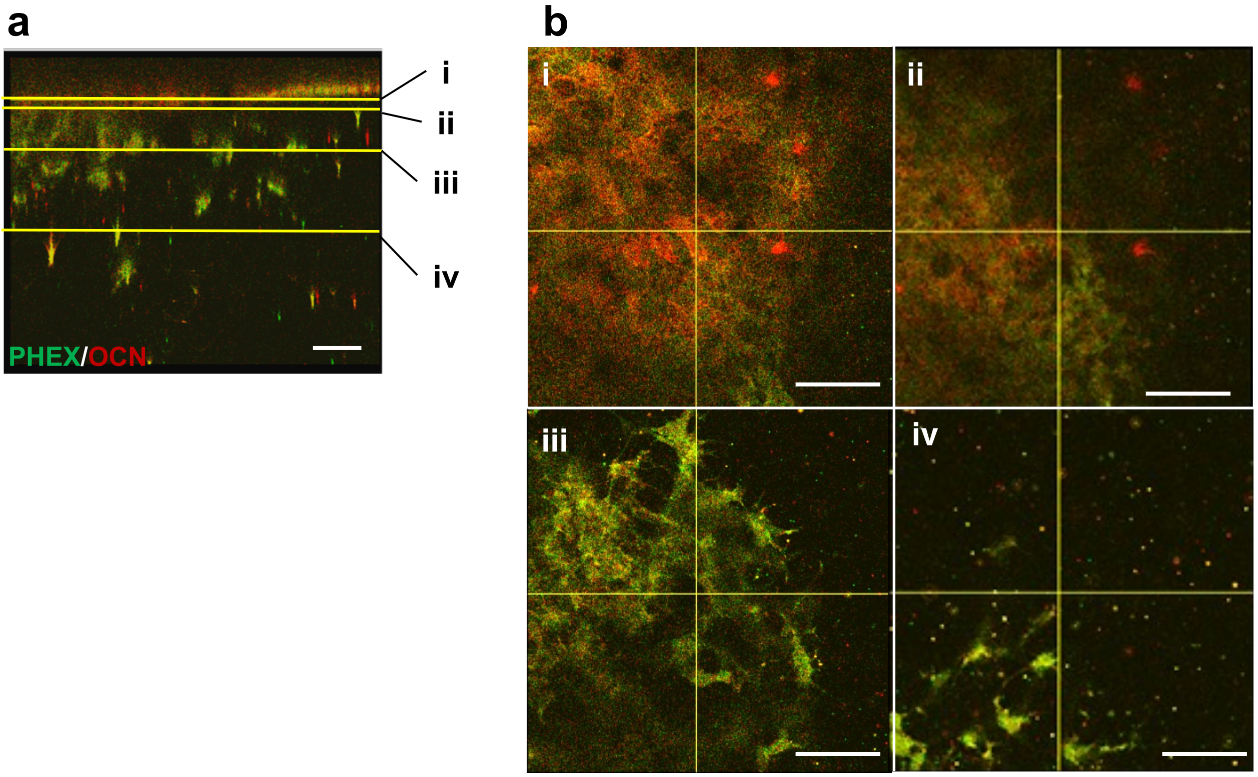

Figure S4. Confocal imaging of osteogenic-induced hiPSCs (409B2) in 3D culture - PHEX and OCN. Immunofluorescence analysis by confocal imaging of vertical (**a**) and horizontal sections (**b**) of 3D culture at day 14. The Roman numerals of the horizontal sections indicates the level taken from the vertical sections. Cells cultured with gel were stained with antibodies for PHEX (green) and OCN (red). Scale bars = 50 µm.

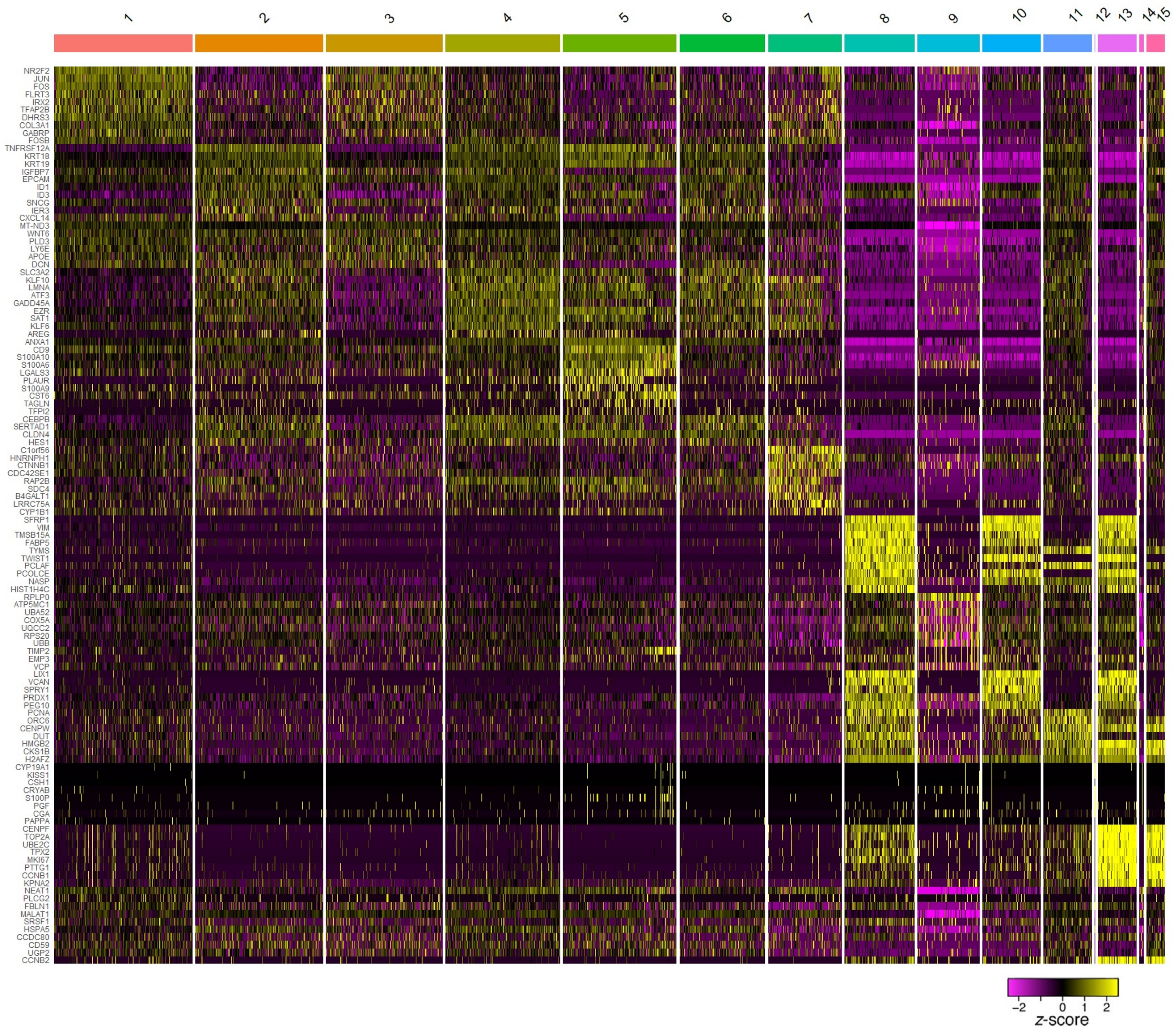

Figure S5. Cluster signature genes. A heatmap showing the the top 10 differentially expressed genes in each UMAP cluster.

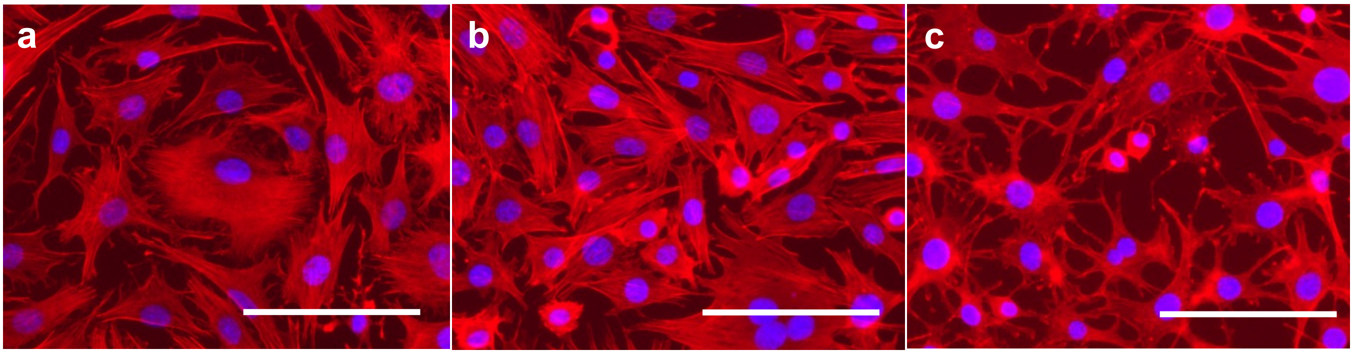

Supplementary Fig. S6. Effects of TGFβ signal on MLO-Y4 cells. MLO-Y4 cells were cultured for 48 hrs in control medium (a), with TGF β1 (5ng/mL) (b), or with SB431542 (10μM). Stained with Phalloidin (red) and DAPI (blue). Scale bars = 100 μm. (b)

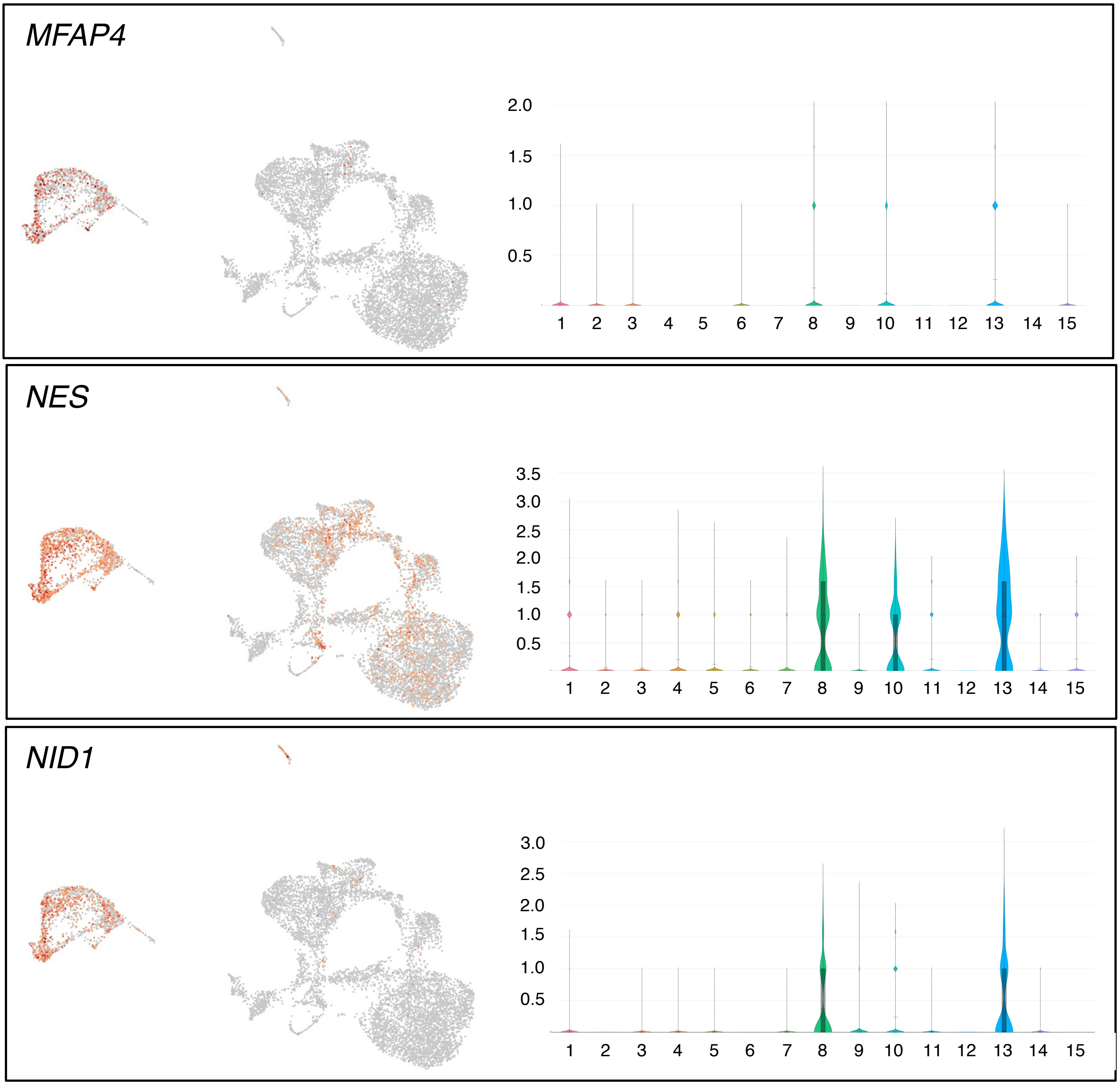

Supplementary Fig. S7. Expression of *MFAP4*, *NE*S, and *NID1*.
Distribution of positive cells were demonstrated in UMAP and the expression level and the amount of positive cells in each cluster demonstrated by Violin plots.

Table S1. Material information

a. Human iPS cell lines

Cell line	Donor (age/gender/site)	Establishment tool	Reference
**414C2**	36 / Female / Skin fibroblast	Episomal plasmid	50
**409B2**	36 / Female / Skin fibroblast	Episomal plasmid	50
**317-12 (201B7-GFP)**	36 / Female / Skin fibroblast (201B7)	Retro virus (201B7)	51

b. Key reagents

**Collagenase**	Wako	9001-12-1	-
**TGFβ1**	Peprotech	100-21C	5 ng/mL

c. Inhibitors

**SB431542**	Selleck Chemicals	S1067	10 μM
**GM6001**	Merck Millipore	CC1010	25 μM

d. Antibodies

**DAPI**	Life Technology	D1306	1:1000
**Collagen Ⅰ**	abcam	ab6308	1:200
**DMP1**	Novus Biologicals	NBP1-89484	1:100
**Phalloidin**	Thermo Fisher Scientific	A22283	1:100
**PHEX**	BIOSS Antibodies	bs-12313R	1:100
**OCN**	R & D system	MAB1419	1:100
**Smad2/3**	CST	#8685	1:100
**pSmad3**	abcam	ab52903	1:100
